# Assessing the Feasibility of Community-Based Colorectal Cancer Screening in Pune, India

**DOI:** 10.1158/2767-9764.CRC-25-0766

**Published:** 2026-05-28

**Authors:** Smita Joshi, Parimal Lawate, B. Kishore Kumar, Pritam Chaudhari

**Affiliations:** 1 Prayas, Amrita Clinic, Pune, India.; 2 Gastroenterology and Liver Clinic, Pune, India.; 3 Consilx, Bangalore, India.

## Abstract

The colorectal cancer rates in India are lower than in other Southeast Asian countries, but there is an increasing trend in incidence as well as mortality. We are reporting the results of a large community-based colorectal cancer screening project implemented through a house-to-house survey. Consenting men and women of ages 50 to 75 years were informed about the purpose of the study and the benefits of colorectal cancer screening. They were educated on how to collect stool sample for occult blood test in a sample collection tube. Health visitors collected the stool sample from the study participants on the next day. Samples were tested with CANCHECK-FOBT, which is Conformite Europeenne-approved and is a rapid, qualitative, two-site sandwich immunoassay for the detection of immunochemical fecal occult blood (iFOB) concentration in human feces. Participants with a positive iFOB test (iFOBT) result underwent colonoscopy as an outpatient procedure at the gastroenterologist’s clinic. A total of 5,003 participants provided consent, and 4,352 (86.98%) provided the stool sample. iFOBT was positive among 70/4,352 (1.61%) participants, of whom 31 (44.92%) completed the colonoscopy procedure. Five participants were diagnosed with adenomatous polyps, and one participant had colorectal cancer. The detection rate of histologically confirmed colorectal cancer was 0.23 and that of adenomatous polyp was 1.15 per 1,000 screened persons. Although the screen positivity and adenomatous polyps and colorectal cancer detection rates in our study are much lower than the in neighboring Southeast Asian countries, these findings highlight the importance of investing in preventive healthcare in India.

**Significance:**

This is the first community-based colorectal cancer screening study from India that suggests the feasibility of colorectal cancer screening. There is an urgent need for investment in prevention of common cancers in the Indian population.

## Introduction

Colorectal cancer is the fourth most common cancer among men and the fifth most common cancer among women in India. The age-standardized incidence rate of colorectal cancer in both sexes combined in India is 4.9 per 100,000 population, and the mortality rate is 2.9 per 100,000 individuals (1). In 2022, approximately 70,000 new colorectal cancer cases and 40,993 deaths were reported in India, and the burden is likely to increase 1.6 times by 2040 (1). Although the incidence and mortality of colorectal cancer is lower in India as compared with other Southeast Asian countries (1), there has been an increasing trend of incidence (2) as well as mortality in India (2, 3). Colorectal cancer is one of the 10 cancers contributing the highest number of disability-adjusted life-years from 1990 to 2016 in India (4).

The National Polyp Study in the United States was a landmark study that provided evidence about 30 years ago that removal of adenomatous polyps from the colon and rectum reduces the incidence of colorectal cancer (5). Subsequently, randomized trials conducted in some developed countries also showed that screening for colorectal cancer, when performed annually or biennially in people ages 50 to 80 years, followed by diagnostic triage with colonoscopy and treatment of detected lesions, is associated with a 15% to 70% reduction in colorectal cancer mortality (6–12). The working group of the International Agency for Research on Cancer on colorectal cancer screening has concluded that there is sufficient evidence that screening with stool-based tests or endoscopic techniques and appropriate management of screen-positives reduces the mortality due to colorectal cancer (13).

Many high-income countries such as the United States, Canada, Australia, New Zealand, most of the countries in Europe, and some countries in the Middle East and West Asia have national colorectal cancer screening programs. Colorectal cancer screening programs aim to reduce the incidence and mortality by detection and removal the dysplastic polyps. Most colorectal cancers occur in preexisting adenomatous polyps in the intestine (14, 15). Colonic polyps, particularly advanced adenomas, may become cancerous and spread to the other areas. A polyp measuring 10 mm or more or showing tubulovillous pattern or severe dysplasia are some of the characteristics defining advanced adenoma (16).

The screening tests for colorectal cancer include colonoscopy, flexible sigmoidoscopy, digital rectal examination, and fecal occult blood test (FOBT). Each of these screening tests has its pros and cons. The most widely used test for colorectal cancer screening is FOBT which detects occult blood in the fecal material (17). Colorectal cancers and polyps sometimes bleed, and FOBT helps to detect tiny amounts of blood in fecal samples. FOBTs are noninvasive, affordable, cost-effective, and acceptable for population screening as they detect intermittent microscopic blood losses from early colorectal cancer to advanced adenomas (18–20).

There are no organized screening programs for colorectal cancer, and nor are there are any reports on community-based colorectal cancer screening in India. We conducted an exploratory, cross-sectional study to assess the feasibility, screening participation, screen positivity, and detection rates of adenomatous polyps and colorectal cancer through FOBT-based screening among the general population in Pune, India.

## Materials and Methods

The study was approved by the Institutional Ethics Committee for Research and was conducted in accordance with the “National Ethical Guidelines for Biomedical and Health Research Involving Human Participants” by the Indian Council of Medical Research (ICMR), 2017 (cited 2026 May 11, at https://ethics.ncdirindia.org/asset/pdf/ICMR_National_Ethical_Guidelines.pdf), which are based on many international guidelines, including the Council for International Organizations of Medical Sciences, Geneva (2002 revised in 2016). The project was implemented in the community setting by a dedicated team consisting of two trained counselors and two trained nurses. The study was implemented in Pune city, primarily among urban and semi-urban populations. The team traveled in a project vehicle equipped with all the necessary stationary and immunochemical FOBT (iFOBT) test kits. Pune Municipal Corporation has divided the city into 15 electoral wards. The study was implemented ward-wise to get representation from all areas of the city. The sequence of the wards was finalized according to convenience. A house-to-house survey was conducted in densely populated urban slums and residential societies, and senior citizen groups were also contacted. The potential participants were given the study information, the importance of colorectal cancer screening, as well as a flyer (approved by the local Ethics Committee), and all eligible individuals in the household were invited to participate in the study.

Apparently healthy, ambulatory men and women of ages 50 to 75 years were given study information by trained counselors which included the importance of colorectal cancer screening and the protocol to be followed if the occult stool blood test was positive. Men and women in sound mental health, without any debilitating physical illness and a prior history of colorectal cancer, were eligible to participate in the study. Participants with a previous history of colorectal cancer or a negative FIT less than 2 years ago were excluded from the study. After the written informed consent form was signed, men and women were interviewed for the personal and demographic data such as age, sex, and family history of colorectal cancer using a structured questionnaire. The CANCHECK-FOBT by Zephyr Biomedicals, India, which is CE-approved, was used for screening. It is a rapid, qualitative, two-site sandwich immunoassay for the detection of fecal occult blood in human feces (21). The participants were provided with a sample collection tube provided by the manufacturer labeled with their name and identification number for stool sample collection. Participants were educated on how to collect stool sample in the sample collection tube. They were asked to collect the stool sample on the next working day in the morning, when the study staff visited either the residence or a predecided point in the community to collect the stool sample. The sample was tested by trained study nurses using the manufacturer’s instructions. The test was reported as “negative” when there was one colored band at the control (C) region, suggesting absence of occult blood or a concentration of occult blood below the detection limit of 200 μg/lit of feces suspension. A positive result was reported when two colored bands appeared at the test (T) and control (C) regions. The test was reported as invalid when no band was visible within 5 minutes. When the test report was invalid, the test was repeated with another test device with the same sample or with a fresh sample on the next day. Participants who tested positive for occult blood in the stool sample were counseled by the trained study staff about further requirement to undergo colonoscopy as an outpatient procedure at the consultant gastroenterologist’s clinic. Once the colonoscopy appointment was confirmed, they were counseled about bowel preparation on the previous day. Participants were provided with travel reimbursement, and the amount was approved by the local Ethics Committee. The maximum distance traveled by the participants for colonoscopy was about 20 km, one way. Colonoscopy was performed under light sedation. Biopsies were performed, and polyps were removed preferably in the same sitting using a wire loop that was passed down the colonoscope and sent for histopathology. Participants with any preexisting medical condition underwent the colonoscopy procedure in a hospital by the same consultant gastroenterologist later. Study participants who were not willing for colonoscopy were contacted telephonically followed by a home visit, and the reasons for noncompliance were noted.

### Statistical analysis

The study primarily employed descriptive statistical methods to quantify participation, screening outcomes, and diagnostic yields. Proportions and percentages were calculated for key indicators such as stool sample return rate, iFOBT positivity, and colonoscopy compliance. Detection rates for colorectal cancer and adenomatous polyps were computed per 1,000 screened participants, providing standardized outcome measures. Differences in iFOBT positivity across demographic subgroups were evaluated using tests for comparison of proportions. To address potential bias due to incomplete colonoscopy follow-up, the authors performed a sensitivity analysis using alternative colonoscopy uptake assumptions.

## Results

**Figure 1.**
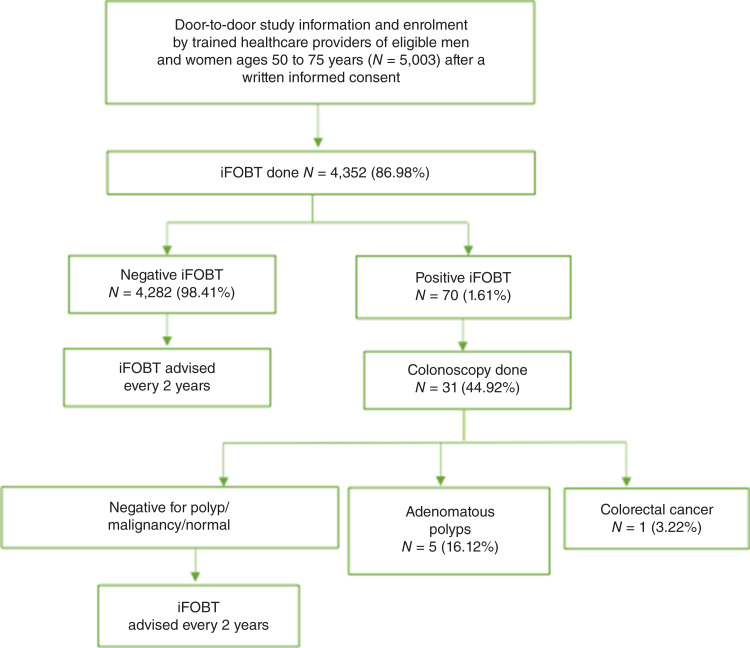
Trial recruitment flowchart.

Participant enrollment was initiated in June 2021 and was completed in December 2022. The last colonoscopy was completed in February 2023. The schematic diagram showing the progress of the study is presented in Fig. 1. A total of 5,003 men and women of ages 50 to 75 years consented for the study, of which 4,352 (86.98%) provided the stool sample for the iFOBT. iFOBT positivity was observed in 1.61% (70/4,352) participants. Those with a negative iFOBT were advised to repeat the test after 2 years. Participants with the positive iFOBT were referred to the study gastroenterologist who performed the colonoscopy. Among the screen-positive participants, 44.92% (31/70) underwent colonoscopy. The histopathology of the excised polyps or biopsies from a suspicious growth detected five participants with adenomatous polyp and one participant with colorectal cancer. The detection rate of histologically confirmed colorectal cancer was 0.23 per 1,000 screened persons, and that of adenomatous polyp was 1.15 per 1,000 screened persons. Participants with adenomatous polyps were advised to follow-up with the gastroenterologist. The participant diagnosed with colorectal cancer was counseled by the gastroenterologist and referred appropriately.

The demographic characteristics and personal history of the study participants are presented in Table 1. Of the 5,003 consenting participants, the majority (*n* = 2,697, 53.9%) were 50 to 59 years old, 1,645 (32.9%) were 60 to 69 years old, and 661 (13.2%) were between 70 and 79 years of age. The mean age was 60.3 years (SD 6.76), and the range was 50 to 78 years. Of the 5,003 consenting participants, 3,219 (64.3%) and 1,784 (35.7%) were females and males, respectively. The other characteristics presented are education, family history of colorectal cancer, body mass index, and any other chronic condition. iFOBT was invalid for 44 of 5,003 The (0.88%) participants, and a repeat test on the same sample on the same day showed valid test results. None of them were iFOBT-positive.

**Table 1.** Characteristic of the participants in the Pune colorectal cancer screening study (*N* = 5,003).

Characteristic	Number	Percent
Age in years	​	​
50–59	2,697	53.9%
60–69	1,645	32.9%
70–79	661	13.2%
Gender	​	​
Female	3,219	64.3%
Male	1,784	35.7%
Education	​	​
Illiterate	1,467	29.3%
Primary and secondary schooling	2,955	59.1%
Intermediate	117	2.3%
University	294	5.9%
Postgraduate degree	170	3.4%
Family history of colorectal cancer	​	​
No	4,986	99.7%
Yes	17	0.3%
Body mass index^a^	​	​
Normal and underweight	2,331	46.6%
Overweight	1,829	36.6%
Obese	843	16.9%
Any other chronic condition	​	​
No	2,832	56.6%
Yes	2,171	43.4%
Key conditions	​	​
Diabetes with or without other chronic condition	998	46%
Hypertension and any other chronic condition	936	43.1%
H/o any other cancer other than colorectal cancer	8	0.4%
Other medical conditions	229	10.5%

a Body mass index classified according to the World Health Organization international classification.

iFOBT positivity by the key demographic characteristics is presented in Table 2. The iFOBT was positive among 1.8% (29/1,575) males and 1.5% females (41/2,777). The overall iFOBT positivity was seen in 1.6% (70/4,352) participants. The test positivity was significantly higher among those with a preexisting chronic condition (45/1,940, 2.3%, 95% CI, 1.7%–3.1%) as compared with those who did not have any preexisting chronic condition (25/2,412, 1%, 95% CI, 0.7%–1.5%), *P* = 0.001. We did not find any significant difference in the other demographic characteristics such as age, urban/rural origin, or family history of colorectal cancer and the iFOBT report.

**Table 2.** Total eligible population, screened population, and test positivity by various key characteristics.

Characteristic	Male					Female					Overall				
	Eligible persons	Number screened with FIT (%)		Number screen-positive (%)		Eligible persons	Number screened with FIT (%)		Number screen-positive (%)		Eligible persons	Number screened with FIT (%)		Number screen-positive (%)	
Overall population	1,784	1,575	88.3%	29	1.8%	3,219	2,777	86.3%	41	1.5%	5,003	4,352	86.99%	70	1.6%
Age (years)	​	​	​	​	​	​	​	​	​	​	​	​	​	​	​
50–59	759	656	86.4%	10	1.5%	1,938	1,653	85.3%	20	1.2%	2,697	2,309	85.6%	30	1.3%
60–69	696	628	90.2%	16	2.5%	949	831	87.6%	13	1.6%	1,645	1,459	88.7%	29	2%
70–79	329	291	88.4%	3	1%	332	293	88.3%	8	2.7%	661	584	88.4%	11	1.9%
Origin	​	​	​	​	​	​	​	​	​	​	​	​	​	​	​
Urban	1,765	1,558	88.3%	28	1.8%	3,156	2,728	86.4%	40	1.5%	4,921	4,286	87.1%	68	1.6%
Rural	19	17	89.5%	1	5.9%	63	49	77.8%	1	2%	82	66	80.5%	2	3%
Family history of colorectal cancer	​	​	​	​	​	​	​	​	​	​	​	​	​	​	​
No	1,778	1,569	88.2%	29	1.8%	3,208	2,767	86.3%	41	1.5%	4,986	4,336	87%	70	1.6%
Yes	6	6	100%	0	0%	11	10	90.9%	0	0%	17	16	94.1%	0	0%
Any other chronic condition	​	​	​	​	​	​	​	​	​	​	​	​	​	​	​
No	1,089	934	85.8%	13	1.4%	1,743	1,478	84.8%	12	0.8%	2,832	2,412	85.2%	25	1%
Yes	695	641	92.2%	16	2.5%	1,476	1,299	88%	29	2.2%	2,171	1,940	89.4%	45	2.3%

The overall colonoscopy attendance among those with iFOBT positivity was 44.3% (31/70; Table 3). Colonoscopy attendance was 58.6% (17/29) and 34.1% (14/41) among men and women, respectively (*P* = 0.04). We did not find any other sociodemographic and medical history factors associated with colonoscopy attendance. Among the two cases suspected to have a cancer on colonoscopy, one was diagnosed with colorectal cancer, and the other had an inflammatory cloacogenic polyp on histopathology. Five adenomatous polyps were diagnosed on histopathology (three in males and two in females). Of the three males with the final diagnosis of adenomatous polyps, two had preexisting diabetes and hypertension, and both the females had hypertension. Among the 31 participants who underwent colonoscopy, 3 of 31 reported bloating and 1 of 31 reported mild cramps. There were no other serious adverse events following colonoscopy.

**Table 3.** Colonoscopy attendance, results, and final diagnosis by gender.

​	Male		Female		Total	
Number screened with FIT	1,575	​	2,777	​	4,352	​
Number screen-positive	29	1.8%	41	1.5%	70	1.6%
Colonoscopy done	17	58.6%	14	34.1%	31	44.3%
Colonoscopy result	​	​	​	​	​	​
Normal	6	35.3%	6	42.9%	12	38.7%
Polyp	3	17.6%	2	14.3%	5	16.1%
Suspected cancer	2	11.8%	0	0%	2	6.5%
Other	6	35.3%	6	42.9%	12	38.7%
Final diagnosis	​	​	​	​	​	​
Normal	7	41.2%	6	42.9%	13	41.9%
Inflammatory (e.g., Crohn’s) disease	1	5.9%	2	14.3%	3	9.7%
Adenomatous polyp	3	17.6%	2	14.3%	5	16.1%
Colorectal cancer	1	5.9%	0	0%	1	3.2%
Others	4	23.5%	4	28.6%	8	25.8%
Ulcerative proctosigmoiditis	1	5.9%	0	0%	1	3.2%

Of the 39 participants who did not visit the gastroenterologist’s clinic for colonoscopy, 32 reported that they were asymptomatic and had fear of colonoscopy and hence refused the procedure, four of them reported having consulted other alternative medicine practitioners or family physicians, two relocated, and the wife of one participant lost her life because of some other cancer and hence he was not willing due to grief.

We conducted sensitivity analyses to examine the robustness of our findings. First, given that only 44.3% of iFOBT-positive participants underwent colonoscopy, we evaluated outcomes under varying compliance assumptions (60% and 80%). The proportional yield of adenomatous polyps and colorectal cancer detection remained fairly consistent, suggesting that our main results were not sensitive to colonoscopy uptake rates.

## Discussion

To our knowledge, this is one of the largest colorectal cancer screening studies conducted in the community setting in India. Screening participation was remarkable among the consenting population in our study. It is encouraging that 87% of the participants provided the stool sample, suggesting that education and awareness among the population increases participation. The screening participation was only 7.14% in another study conducted in India that used colonoscopy for screening (22). Overall, the screening participation for colorectal cancer has remained low in many East Asian countries (23). Lack of awareness often leads to most patients with colorectal cancer being diagnosed at an advanced stage with a poor prognosis. The screen positivity in our study was 1.6%, lower than that reported from many other countries such as Morocco (4.7%; ref. 24), France (2.6%; ref. 25), Malaysia (10%; ref. 26), and the United States (12.6%; ref. 11). In Morocco, the detection rates of advanced adenomas and colorectal cancer were 4 in 1,000 and 0.5 in 1,000 individuals screened, respectively, in contrast to the lower detection rates of advanced adenomas (1.15 per 1,000 individuals) and colorectal cancer (0.23 per 1,000 screened persons) in our study.

For a screening program to be effective and achieve mortality reduction, adherence to the diagnostic evaluation of the screen-positive participants is necessary. The colonoscopy attendance in our study was modest, and the majority of the participants who refused colonoscopy mentioned that they were asymptomatic and were afraid of colonoscopy. As it is difficult for the lay people to understand the need of the screening while being asymptomatic, it is important that clear messages are conveyed to the people participating in screening programs. Some of the factors associated with nonattendance at colonoscopy screening from a previous study in the United Kingdom include other commitments, unwillingness, a feeling that the FOBT was a false positive, another health issue taking priority, failing to complete bowel preparation, practical barriers (e.g., lack of transport), and having had planned colonoscopy elsewhere (27).

Although the adherence rate to colonoscopy after the positive iFOBT was low (45%) in our study, our sensitivity analysis to examine the robustness of our findings showed that the proportional yield of adenomatous polyps and colorectal cancer detection remained fairly consistent, suggesting that our main results were not sensitive to colonoscopy uptake rates. The colonoscopy participation rate was significantly higher in men than in women in our study. High compliance with colonoscopy can have a strong clinical impact on colorectal cancer mortality (28, 29). Some of the determinants of compliance described earlier include age less than 59 years, higher education, female sex, and nonmanual work (29).

We used the iFOBT (also known as FIT) in our study. Currently, two types of FOBTs are available: guaiac FOBT (gFOBT) and iFOBT. gFOBT uses the chemical guaiac to detect heme, the iron-containing component of the blood protein hemoglobin, and iFOBT uses antibodies to detect globin, the protein of human hemoglobin in feces. Dietary restrictions and changes, such as avoiding meat, certain vegetables, vitamin C, iron supplements, and aspirin, as well as increasing fiber consumption, are not necessary for iFOBT-based screening, and therefore it is more convenient.

Some of the challenges for effective cancer control in India are affordability, adequate numbers of trained healthcare personnel, and sociocultural barriers (30). The rising burden of cancers in India and high mortality due to late-stage diagnosis calls for planning effective risk reduction strategies such as reducing consumption of red meat and processed meat (31), consumption of alcohol (32), body fatness (33), and tobacco smoking (33). Once colorectal cancer is diagnosed, out-of-pocket, catastrophic expenditure for treatment in India has already been reported (34). The World Health Organization recommends that screening programs should be implemented only when the disease burden is high enough to justify the effort and cost, the health system has the resources to reach the target population, diagnostic and treatment services are in place for those with abnormal results, and the effectiveness of screening has been demonstrated (35, 36). The first meeting by the International Cancer Screening Network was held in India in September 2016, and a summary report with key recommendations that list some of the important elements of population-based cancer screening programs has already been published (37). Our study has demonstrated the feasibility of screening men and women for colorectal cancer in the community setting; however, the compliance to colonoscopy screening needs to be improved with additional strategies, such as widespread community awareness and education.

Colorectal cancer incidence rates vary across the world, and the rates are lowest in Central Asia, West Asia, and South Asia (17). The colorectal cancer incidence rate in India is lower than in other neighboring countries in Southeast Asia (17). Screen positivity for occult blood was also lower in our study as compared with that in other neighboring countries. It is possible that the lower colorectal cancer incidence rate in India is reflected in the lower screen positivity rates; however, it is important to replicate the study in different populations in India to document estimates of screen positivity and disease detection rates and also to evaluate cost-effectiveness based on the data obtained from the screening projects. At the same time, considering the fact that the adenomatous polyp detection rates were higher among those with preexisting medical conditions, this group may be targeted by treating physicians.

The population of Pune city according to the last Census of India in 2011 is 3,304,888 [Government of India (Internet); cited 2026 March 11; available at https://censusindia.gov.in/census.website/data/population-finder]. Although the study population represents 0.1% of the total population of Pune city, this exploratory study provides initial evidence on the feasibility of community-based colorectal cancer screening, including screening participation, test positivity, and detection rates of adenomatous polyps and colorectal cancer using iFOBT in the general population of Pune, India.

Screening of the population for colorectal cancer is worthwhile when the health benefits outweigh the cost (17). Although the colorectal cancer and adenomatous polyp detection rates in our study are not as high as the rates seen in developed countries or other South Asian countries, the definite advantages of organized cancer screening programs for reducing mortality and the rising burden of cancers in India make a strong case for vertical investment in screening for cancers such as cervical, breast, and colorectal cancers.

## Data Availability

The data that support the findings of this study are available from the corresponding author upon reasonable request. The study was approved by the Institutional Ethics Committee for Research of Prayas, a nonprofit organization in Pune, India. The study has been registered on the Clinical Trials Registry of India (CTRI), and the CTRI number is CTRI/2021/06/034346. All enrolled participants signed a written informed consent form.
